# High-Precision, Whole-Genome Sequencing of Laboratory Strains Facilitates Genetic Studies

**DOI:** 10.1371/journal.pgen.1000139

**Published:** 2008-08-01

**Authors:** Anjana Srivatsan, Yi Han, Jianlan Peng, Ashley K. Tehranchi, Richard Gibbs, Jue D. Wang, Rui Chen

**Affiliations:** 1 Department of Molecular and Human Genetics, Baylor College of Medicine, Houston, Texas, United States of America; 2 Human Genome Sequencing Center, Baylor College of Medicine, Houston, Texas, United States of America; Stanford University, United States of America

## Abstract

Whole-genome sequencing is a powerful technique for obtaining the reference sequence information of multiple organisms. Its use can be dramatically expanded to rapidly identify genomic variations, which can be linked with phenotypes to obtain biological insights. We explored these potential applications using the emerging next-generation sequencing platform Solexa Genome Analyzer, and the well-characterized model bacterium *Bacillus subtilis*. Combining sequencing with experimental verification, we first improved the accuracy of the published sequence of the *B. subtilis* reference strain 168, then obtained sequences of multiple related laboratory strains and different isolates of each strain. This provides a framework for comparing the divergence between different laboratory strains and between their individual isolates. We also demonstrated the power of Solexa sequencing by using its results to predict a defect in the citrate signal transduction pathway of a common laboratory strain, which we verified experimentally. Finally, we examined the molecular nature of spontaneously generated mutations that suppress the growth defect caused by deletion of the stringent response mediator *relA*. Using whole-genome sequencing, we rapidly mapped these suppressor mutations to two small homologs of *relA*. Interestingly, stable suppressor strains had mutations in both genes, with each mutation alone partially relieving the *relA* growth defect. This supports an intriguing three-locus interaction module that is not easily identifiable through traditional suppressor mapping. We conclude that whole-genome sequencing can drastically accelerate the identification of suppressor mutations and complex genetic interactions, and it can be applied as a standard tool to investigate the genetic traits of model organisms.

## Author Summary

In this manuscript, we describe novel applications of the newly developed Solexa sequencing technology. We aim to provide insights into the following questions: (1) Can whole-genome sequencing, while rapidly surveying mega-bases of genome information, also reliably identify variations at the base-pair resolution? (2) Can it be used to identify the differences between isolates of the same laboratory strain and between different laboratory strains? (3) Can it be used as a genetic tool to predict phenotypes and identify suppressors? To this end, we performed whole-genome shotgun sequencing of several related strains of the widely studied model bacterium *Bacillus subtilis*, we identified genomic variations that potentially underlie strain-specific phenotypes, which occur frequently in biological studies, and we found multiple suppressor mutations within a single strain that are difficult to discern through traditional methods. We conclude that whole-genome sequencing can be directly used to guide genetic studies.

## Introduction

Completion of the whole-genome sequencing of many organisms, ranging from bacteria to humans, has transformed the way in which biological research is conducted. Genome sequencing is mostly used as a resource to obtain the reference sequence information of laboratory species, and its full applications in genetic research remain unexplored, due to its time-consuming and expensive nature. These problems can potentially be circumvented using next-generation sequencing platforms such as 454, Solexa, and SOLiD, which perform cost effective, high throughput sequencing, thus making sequencing of individual isolates a feasible option. For example, with the Solexa platform, a large number of DNA fragments are immobilized on a solid surface and read with fluorescence-labeled nucleotides simultaneously. Millions of 36–50 base pair long reads can be obtained from each sample lane at a cost of less than $1000. The deep sampling of DNA fragments allows rapid procurement of high coverage genome sequence information. These new, powerful sequencing technologies will be widely accessible in the near future, and have the potential to revolutionize the way in which current research is conducted.

Genetic studies with model organisms are often conducted with multiple laboratory strains without detailed information on how these strains differ from one another. The observation of several ‘strain-specific phenotypes’ suggests underlying differences in their genomes. Further, multiple isolates are used for each strain, in most cases, without an objective measure of their isogenicity. Direct sequencing has the potential to identify such unknown differences to inform experimental design and analysis, and reveal avenues for reverse genetic studies. Another potentially tremendous benefit from the knowledge of complete and precise genome sequences is the direct identification of suppressor mutations. Traditional genetic mapping to identify suppressors is a time-consuming process, which can be further complicated by unstable strains, dominant alleles, and multiple suppressors occurring in a single strain. Epistatic interactions are commonly studied between pairs of relevant genes and suppressor mapping is often designed to reveal two-locus genetic interactions. Despite the potential prevalence of multi-component genetic interactions in organisms, they are difficult to identify with traditional genetic approaches. Whole-genome sequencing, however, can circumvent these difficulties, by identifying multiple mutations in a given strain in a single step.

The Gram-positive bacterium *Bacillus subtilis* is an ideal system for a ‘proof-of-principle’ study of the applications of whole-genome sequencing. Being an excellent model for investigating the mechanisms of gene regulation, differentiation, and metabolism, *B. subtilis* has been extensively studied in hundreds of laboratories world-wide for more than half a century using a variety of laboratory strains [1],[2]. However, the laboratory strain 168 [3] is the only *B. subtilis* strain with known genomic sequence, obtained through an extensive collaboration more than ten years ago [4]. 168 was generated by mutagenic X-rays and UV treatment of the wild type *B. subtilis* (Marburg) strain [2], resulting in the requirement for externally added tryptophan for growth, and the inability to produce a secreted antibiotic surfactin, due to mutations in the genes *trpC* and *sfp*, respectively [5]. Another broadly studied strain JH642 [6] which was obtained by multiple gene exchange experiments ([7], and James Hoch, personal communication) further differs from 168, including mutations in the genes *pheA* and *ilvB* that lead to phenylalanine requirement and cold sensitivity, respectively [8]. On the other hand, some laboratory strains (such as NCIB 3610 and SMY) do not have these phenotypes and are proposed to be true wild type strains. Thus, obtaining the genome information of these different laboratory strains and their independent isolates would aid in understanding of the reproducibility of results between strains, the molecular bases of strain-specific phenotypes, as well as defining the ‘isogenicity’ of isolates.

**Figure 1:**
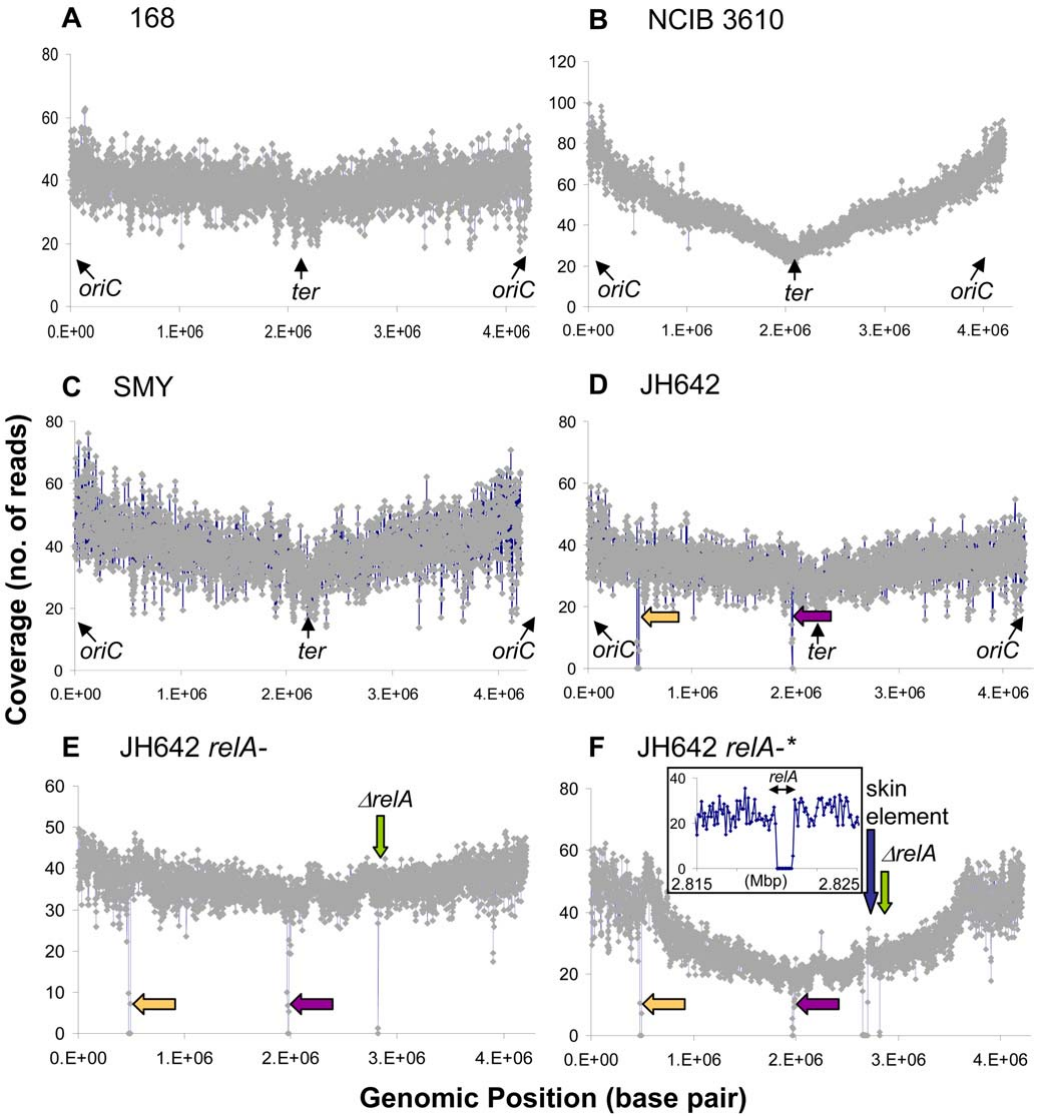
*B. subtilis* strains sequenced and the number of Solexa sequencing reads at each genomic position for each strain. A) *B. subtilis* 168 (BGSC 1A700); B) NCIB 3610 (BGSC 3A1); C) SMY (BGSC 1A775); D) JH642 (BGSC 1A96); E) JH642 with *relA* deletion; F) JH642 with *relA* deletion and second site suppressors (*relA*-*). Yellow arrows: 18 kb deletion (*ydzA*- *ydbA*) in JH642; purple arrows: 9 kb deletion (*ppsC*- *ppsD*) in JH642; green arrows: 2 kb *relA* deletion in strains *relA*- and *relA*-*; blue arrow: skin element deletion in *relA*-*. Coverage was obtained by calculating the average number of reads in each 1 kb window. Inset in Figure 1F) is the high resolution coverage map (average reads per 0.2 kb window) of the *relA* region. The origin of replication is at the 0/4.2 Mbp genomic position, and the terminus of replication is at the 2.1 Mbp genomic position. NCIB 3610 and *relA*-* were grown to mid log phase, while the other strains were grown to stationary phase. The different shapes of the coverage maps might be partly due to these differences in growth phases.

In this work, we used the Solexa Genome Analyzer method to sequence the related laboratory strains 168, NCIB 3610, SMY and JH642 (Figure 1). Based on our results, we provide an updated draft of the 168 reference sequence. In addition, we found that independent isolates of the same strain differ by as few as 6 base pairs, while the difference between laboratory strains is larger. We verified multiple genome variations reported in the literature, and verified selected additional base variations by Sanger sequencing. Further, by correlating the genotypes with the phenotypes, we experimentally uncovered a hidden phenotype of the laboratory strain JH642 due to a defect in its two-component histidine kinase sensor responsible for citrate import. Finally, we identified the multiple causal nucleotide alterations in a single suppressor strain of a *relA* deletion mutant. The RelA enzyme is crucial for modulating the level of the small nucleotide (p)ppGpp, which is central in mounting the bacterial starvation response-the stringent response [9]. We identified mutations in two small homologs of *relA* that were independently shown to have (p)ppGpp synthesis activities [10],[11] and found that mutations in each of these genes lead to partial suppression of the *relA*-associated growth defect. As a result, multiple types of suppressor mutations are generated in these genes in response to deletion of *relA*, making their identification difficult with traditional genetic mapping. Hence whole-genome sequencing enables the identification of individual nodes of multi-component genetic interaction networks simultaneously, and maps evolutionary pathways that can promote the growth of a genetically compromised strain. Our results offer strong proof that the Solexa method can be used to rapidly reveal multiple aspects of genomic content and organization, especially base substitutions, which greatly simplifies experimental design and facilitates our understanding of the biology of model organisms. This method can be applied broadly, including to similar studies with other bacterial and higher organisms [12].

## Results

### Whole-Genome Shotgun Re-Sequencing of *B. subtilis* 168

Whole-genome sequencing using newly developed high-throughput shotgun methods represents a potentially powerful tool for many studies conducted in the laboratory. To explore the utility of this new technology, we first tested the accuracy of the sequences generated by the Solexa Genome Analyzer by re-sequencing the ∼4.21 million base pair long reference genome of *B. subtilis* strain 168 (BGSC 1A700, accessioned directly to the Bacillus Genetic Stock Center (BGSC) from Anagnostopoulos C), which was an isolate whose sequence was first obtained 10 years ago (Zeigler D, personal communication) [4].

Genomic DNA was extracted from *B. subtilis* 168 and whole-genome shotgun sequences were obtained using the Genome Analyzer (see Materials and Methods), and a total of 5.29 million 36 base pair long reads were generated. We first mapped these reads to the published *B. subtilis* reference genome (Genbank entry AL009126) using the MAQ (Mapping and Assembly with Qualities) software (http://maq.sourceforge.net/index.shtml) (Heng Li, Richard Durbin, personal communication) to generate a consensus sequence. 4.63 million reads could be mapped to the reference genome, equivalent to an average of 39.6-fold sequencing coverage across the entire genome (Figure 1A and Table 1). At each base, a quality score was generated based on the reads by the statistical model of MAQ. 98.8% (4.16/4.21 Mb) of all consensus bases had a quality score of 40 (estimated error rate <10^−4^) or higher, and >99.5% of all consensus bases had a quality score of 30 (estimated error rate <10^−3^) or higher. After evaluating the quality score with empirical methods (outlined below), we chose to use the quality score of 40 as the cut off since at this score the vast majority of reads agree with the consensus. This score was used for all the subsequent sequencing described below.

**Table 1:** Summary of sequence reads and coverage.

Strain names	168	168	SMY	SMY	NCIB3610	JH642	JH642	JH642 relA-*	JH642 *relA-*
BGSC accession no.	1A700	1A1	1A775	1A868	3A1	1A96	1A867		
Total Reads (million)	5.23	6.13	4.64	6.59	6.68	5.14	2.5	4.08	4.68
Mapped Reads (10^6^)	4.63	5.82	3.99	6.22	5.91	4.78	2.42	3.83	4.41
Coverage (fold)	38.5	48.7	33.2	51.7	49.0	39.7	20.7	31.2	36.1

Among bases with a quality score of 40 or higher, 1519 bases were found to be different from the published reference sequence. There are three possibilities that can account for these discrepancies: first, these are errors in our current sequence; second, these are errors in the published reference sequence; third, these reflect differences between the independent isolates of 168. Since the Solexa sequencing platform was developed recently, we first evaluated the quality of the sequence data generated by this platform.

**Figure 2:**
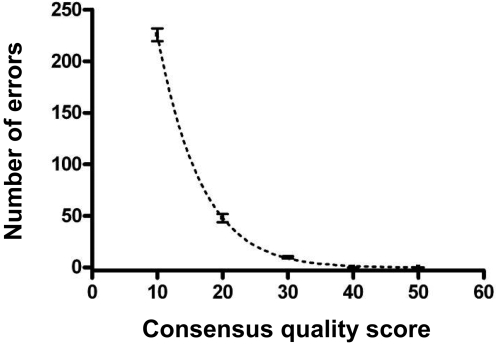
Quality analysis of sequences generated with the Illumina/Solexa platform. Sequencing errors were estimated by self-against-self comparison tests. Independent Solexa reads from a single 168 sample were randomly shuffled and split into two equal groups. Consensus sequences from these two groups were compared, and the experiment was repeated 10 times to calculate the average number of errors. The observed average number of errors decreased exponentially with the consensus quality score calculated by MAQ.

**Figure 3:**
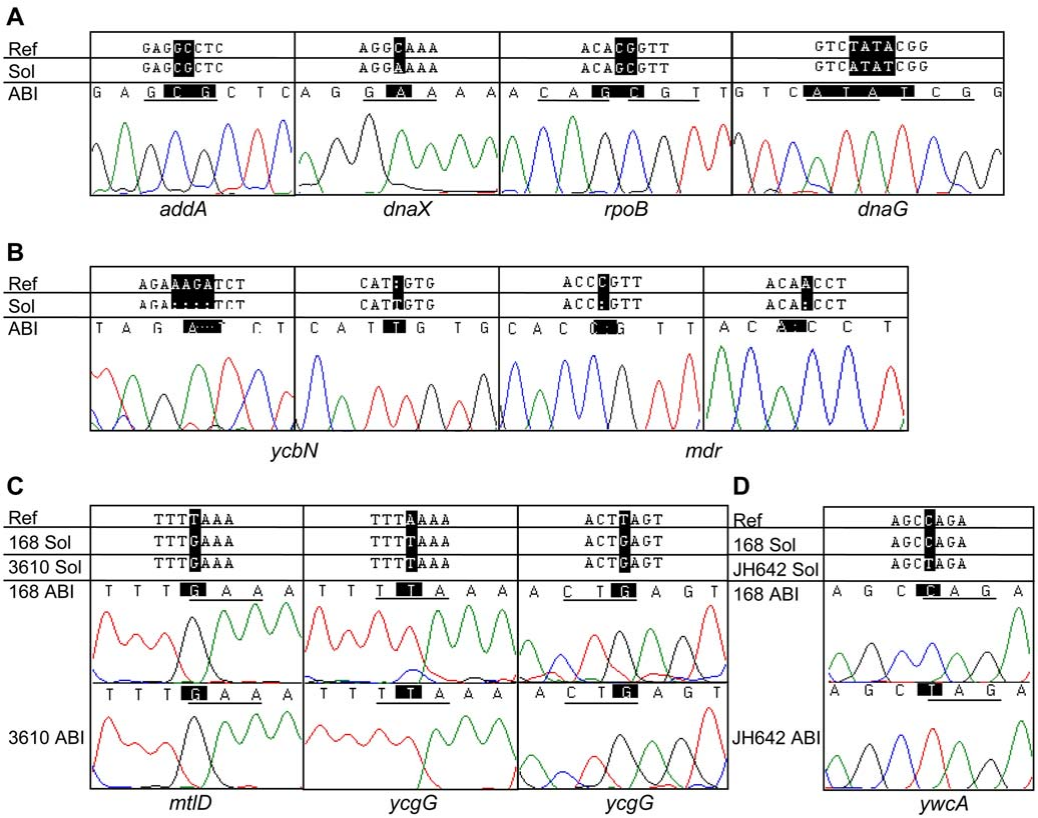
Verification of the Solexa results by Sanger sequencing. A) Selected reads from strain 168 where results from Solexa suggest missense changes with respect to the reference. B) Selected reads from strain 168 where results from Solexa suggest insertions or deletions with respect to the reference. C) Selected reads from 168 and NCIB 3610 where results from Solexa suggest nonsense mutations in the reference sequence. D) Selected reads from JH642 and 168 where results from Solexa suggest nonsense changes in JH642 with respect to 168. In each case, the published reference sequence is indicated first, the Solexa sequence(s) are shown next, and the Sanger sequencing results and corresponding chromatograms are depicted last.

Multiple independent methods were used to estimate the accuracy of the *B. subtilis* 168 genomic sequence generated by the Solexa platform. First, sequencing controls were used to measure the sequencing error rate. To obtain an empirical error rate, a previously sequenced 170 kb BAC (bacterial artificial chromosome) clone (bCX17K10_79963.162948_bCX98J2_1) was re-sequenced using the Genome Analyzer. By comparing the consensus sequence to the BAC reference sequence, we found that the error rate was around 0.01% at a quality score of 40, meaning that the accuracy of each base call was around 99.99% (data not shown). This is consistent with the idea that the quality score calculated by MAQ is similar to the phred quality score which is defined as −10×log_10_error (error is defined as probability that the base is called wrong) [13]. Second, we performed self-against-self comparison experiments with the sequences of 168 generated by the Genome Analyzer. We reasoned that consensus sequences obtained from the same homogenous sample should be identical if there are no random sequencing errors. Therefore, differences observed between consensus sequences derived from two independent groups of reads from the same sample can provide a reasonable estimation of the error rate. Reads from the strain 168 were randomly shuffled and then split equally into two groups. Consensus sequences derived from these two groups of reads were compared and the number of varying bases was obtained. A total of 10 random shuffle experiments were conducted on the sequencing reads obtained for the *B. subtilis* strain 168. An average of eight sequencing errors (out of the entire 4.21 Mb genome) were observed at a cutoff score of 30 while no errors were found at a cutoff score of 40 (Figure 2). Therefore, we used a quality cutoff score of 40 to minimize sequence errors in our current 168 sequence. Third, eight randomly selected discrepant regions with a quality score of 40 or higher were cross-validated by the Sanger sequencing platform. We found that for all eight regions, results obtained by the Sanger method were consistent with the Solexa reads (Figure 3A and results not shown).

In addition to the base substitutions, we noticed that 1725 bases of the reference sequence fell into gaps based on the MAQ consensus sequence. Since MAQ uses un-gapped alignments to map reads to the reference sequence, it is possible that these sequence gaps occurred due to variations between the reference sequence and the reads, rather than due to lack of sequence coverage. To further investigate this possibility, we used two independent methods. First, all Solexa reads were mapped iteratively to the reference genome, allowing gaps and up to 3 mismatches, using the SOAP (Short Oligonucleotide Alignment Program) algorithm [14]. The consensus sequence was deduced from the reads that mapped to sequence gaps by selecting the majority call. The SOAP algorithm also minimizes base calling errors near alignment gaps. However, one limitation of aligning reads to the reference is that it is difficult to align regions that contain many variations or with large insertions or deletions. In several places, a cluster of variations was found where no reads could be aligned. To circumvent this problem, we performed de novo assembly using the Edena (Exact De Novo Assembly) software, which is based on the classical contig assembly approach [15]. A total of 5277 contigs were obtained with total size of more than 3.85 Mb, accounting for about 90% of the genome. These contigs were then aligned to the reference genome using the *Crossmatch* program, to determine the sequences within the gaps. Consistent with our prediction, these sequence gap regions were highly enriched in variations, and we found an additional 278 base substitutions within them.

Using a combination of de novo assembly with the alignment approaches, we significantly reduced the sequence gaps for strain 168 to only 191 bases. Closer examination of these gap regions indicated that they were mainly localized at the 460957 and 1018364 genomic positions. Gaps at these two regions are likely due to a high degree of divergence, which prevents the proper alignment of the contigs. Further experiments will be needed to test this possibility. Finally, to identify additional substitutions as well as insertions and deletions in the genome, we further examined the alignment between the Solexa reads, the de novo assembly, and the reference sequence. Only those insertions and deletions that were consistently identified in both the Solexa reads and the de novo assembly alignments were considered to be true. To test our result, two regions were selected and tested by Sanger sequencing and both regions were confirmed to match the Solexa sequence (Figure 3B). As a result, 82 insertions and 85 deletions were included in our revised reference genome sequence.

Finally, we sequenced an independent isolate of 168- BGSC 1A1 for comparison. This isolate was accessioned in the mid 1970s from James Shapiro and was subsequently used by the Japanese members of the sequencing consortium [4] (Zeigler D, personal communication). Only 31 differences between these two isolates of 168 were identified by Solexa (Table S1). Therefore, we modified the 168 reference sequence [4] by updating it with the high quality reads from our sequencing of the 168 isolate BGSC 1A700. This whole genome shotgun project has been deposited at DDBJ/EMBL/GenBank under the project accession number ABQK00000000. The version described in this paper is the first version, ABQK01000000.

### Direct Shotgun Sequencing to Identify the Genomic Divergence of the Laboratory Strains JH642, SMY, and NCIB 3610

The comparison of two independent isolates of 168 revealed a small amount of heterogeneity between them. To evaluate the divergence between other independent laboratory strains and their isolates, we further compared the sequences of several related *B. subtilis* laboratory strains. These are the prototrophic strains NCIB 3610 [1] and SMY (subtilis-Marburg-Yale) [16], and the widely used laboratory strain JH642 created by James Hoch [6],[7]. In particular, we sequenced the isolate of NCIB 3610, BGSC 3A1 (accessioned from the NCIB collection in England 30 years ago), two independent isolates of the strain SMY (one was obtained from A. Sonenshein and later deposited as BGSC 1A868, and the other was BGSC 1A775), and two independent isolates of the laboratory strain JH642 (one was BGSC 1A96, accessioned from James Hoch, and the other was obtained from A. Grossman and later deposited as BGSC 1A867) [17],[18] (Table 2). We assembled the sequences with MAQ, SOAP and Edena as described for 168. The whole-genome shotgun sequences were deposited at DDBJ/EMBL/GenBank under the project accessions ABQL00000000 (NCIB 3610; BGSC 3A1), ABQN00000000 (SMY; BGSC 1A775) and ABQM00000000 (JH642; BGSC 1A96). The versions described here are the first versions ABQL01000000, ABQN01000000 and ABQM01000000, respectively.

**Table 2:** *B. subtilis* strains used.

Strains	Genotype, Source and Reference
168	*trpC2*, BGSC 1A700 [4]
168	*trpC2*, BGSC 1A1 [4]
SMY	prototroph, BGSC 1A775 [16]
SMY	prototroph, BGSC 1A868, A.L. Sonenshein [16]
NCIB 3610	prototroph (BGSC 3A1) [1]
JH642	*trpC2 pheA1*, BGSC 1A96 [7],[18]
JH642	*trpC2 pheA1*, BGSC 1A867, A.D. Grossman [7],[18]
JDW441	JH642 *pheA*+ (this study)
JDW442	JH642 *trpC*+ *pheA*+ (this study)
JDW522	JH642 *trpC*+ *pheA*+ *citS*+ (this study)
TW30 (*relA-*)	JH642 *relA*::*mls* [30]
JDW162 (relA-*)	JH642 *relA*::mls yjbM* ywaC* (this study)
JDW506	JH642 yjbM* (this study)
AS021	JH642 ywaC* (cm^R^) (this study)
AS012	JH642 *amyE*:: *Pspac*(hy)_*yjbM (spec^R^)* (this study)
AS013	JH642 *amyE*:: *Pspac*(hy)_*ywaC (spec^R^)* (this study)

We found that different isolates of the same strain in general have very few nucleotide differences. The two isolates of SMY differed by only 13 bases (Table S2) and the two isolates of JH642 differed by only 6 bases (Table S3). These base differences might include true differences between the isolates and any additional differences introduced when we chose a single colony of each isolate for sequencing.

Between different strains, the number of differences was variable. Using the MAQ program at a quality score cutoff of 40, we identified 41 base differences between the chromosomal sequences of NCIB 3610 (BGC 3A1) and 168 (BGSC 1A700). We verified selected regions by Sanger sequencing (Figure 3C). In addition, by performing de novo assembly of the Solexa reads from the NCIB 3610 strain, we found that about 78.3 kb of sequence did not map to the reference genome. It was previously reported that a large (∼80 kb), low copy-number plasmid exists in NCIB 3610 [19]. Indeed, when used to query the GenBank nucleotide sequence database, this 78.3 kb sequence did not yield any significant hits, except for a 7.2 kb region which had 97% overall identity with the region of replication of the plasmid pLS32 isolated from *B. natto* [20]. These data strongly suggest that the unmapped sequences in our reads of NCIB 3610 were from a plasmid which is absent from SMY, 168 and JH642. The draft sequence of this plasmid (named pAS32) was thus merged into one file consisting of multiple contigs (accession ABQL00000000).

**Figure 4:**
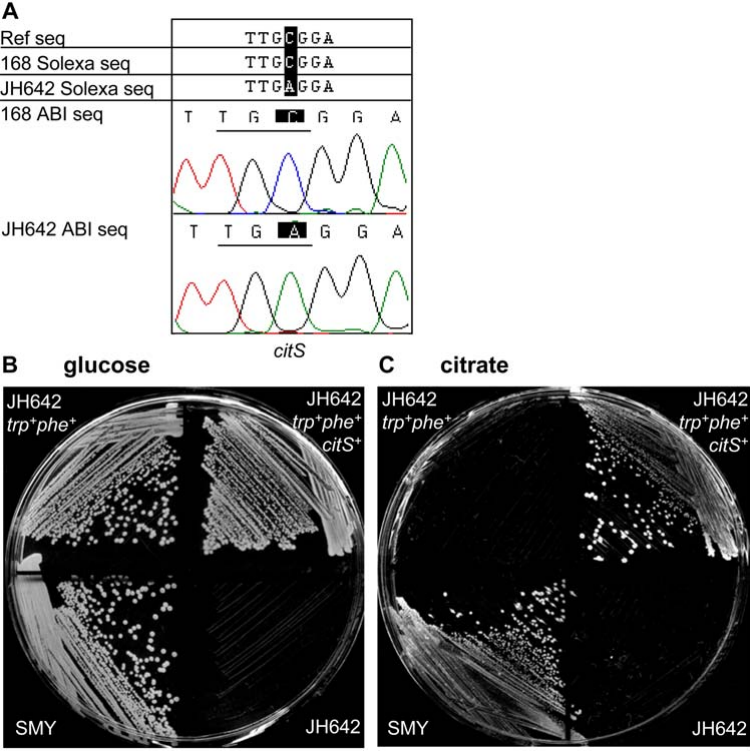
Verification that the nonsense mutation in *citS* leads to a defect in the citrate import pathway. A) Sanger sequencing verification of the Solexa *citS* sequence. Sequences are shown in the following order: the published reference sequence first, followed by the Solexa results for the *citS* gene in strains 168 and JH642, and the Sanger results for both strains shown last. B, C) Rescue of the *citS-* phenotype of JH642. Strains were plated to obtain single colonies on minimal media supplemented with glucose (B) or citrate (C) as the sole carbon source. The strains shown are: the prototroph SMY, the lab strain JH642, a JH642-derived strain that is ‘cured’ of its growth-dependence on the amino acids tryptophan and phenylalanine (JH642 *trp^+^*, *phe* ^+^), and finally, the latter strain, transformed with the complete *citS* gene from SMY (JH642 *trp^+^ phe^+^ citS^+^*). JH642 *trp^+^ phe^+^* is able to utilize glucose but not citrate. JH642 *trp^+^ phe^+^ citS^+^* is able to utilize both glucose and citrate.

Using MAQ at the quality score cutoff of 40, we identified 27 base differences between SMY (BGSC 1A775) and 168 (BGSC 1A700), in addition to a region of highly clustered mutations (2373412-2379802 bp). We identified 47 base differences between JH642 (BGSC 1A96) and 168 (BGSC 1A700), in addition to a short 4 kb region from *sacA* to *ywcI* (3902573-3906848 bp) with highly clustered mutations. These clusters were most likely introduced by gene transfer (see Discussion). The base differences between JH642 and 168 included several known mutations such as those in the gene *ilvB* [8], and a single T to C change at base 704 of *pheA* encoding prephenate dehydratase, which leads to the missense mutation S235L (TTA ->TCA). The latter mutation accounts for the phenylalanine auxotrophy of JH642 and it can be rescued by transforming JH642 with the wild type *pheA* gene (in the form of a purified PCR product) (data not shown). To further verify our sequencing results, regions around 8 of the bases that were found to be different in JH642 and 168 by Solexa were amplified from both strains by PCR and sequenced using the Sanger method. All the eight regions were found to be the same as our JH642 Solexa results, and different from 168 (Figure 3D, 4A and data not shown), demonstrating the high accuracy of the Solexa method for detecting base substitutions. In addition, examination of the sequence coverage across the JH642 genome revealed two deleted regions (Figure 1D). One region was a cluster of 18 kb between 475,381 bp and 491,816 bp (from *ydzA* to *ydbA*). This deletion was identified previously [21]. This region includes genes such as *lrpC* [22], *topB* [23],[24] and *mutT* [25],[26], which are proposed to be important for DNA recombination, topology and replication fidelity, respectively. The second deleted region was ∼9 kb between 1,967,180 bp to 1,977,755 bp, encoding *ppsC*-*ppsD*. These genes are responsible for synthesizing plipastatin, a fungicidal lipopeptide [27], and therefore there was no evolutionary pressure to maintain them within the laboratory strain, perhaps contributing to their loss.

### Genomic Differences Can Be Used to Predict Phenotypic Differences

We experimentally tested whether the base variations we identified by Solexa sequencing had phenotypic consequences. We observed two nonsense mutations in JH642, within the genes *citS* and *ywcA* (Figure 4A). *ywcA* encodes a putative symporter with an unknown function. *citS* encodes a component of a signal transduction system that regulates the expression of a Mg^2+^-citrate transporter. A *citS*-deficient strain is unable to grow on minimal media plates that have citrate as the sole carbon source [28]. The nonsense mutation we identified in *citS* occurs near the 3′ end of the gene and we examined whether this mutation might lead to a null phenotype in JH642. One complication is that JH642 cannot grow without the amino acids phenylalanine and tryptophan, which can also act as carbon sources, thus perhaps masking any potential citrate utilization defect. Therefore, we first performed gene replacement in JH642 to render it capable of growing in the absence of these two amino acids. This was done by consecutive transformation of JH642 with the PCR amplicons of the wild type *pheA* and *trpC* genes (from NCIB 3610), and selection on minimal media plates with glucose as the sole carbon source. Thus we obtained a JH642-derived strain cured of its tryptophan and phenylalanine auxotrophies. We found that this strain was able to grow on minimal plates with glucose, but not citrate, as the sole carbon source (Figure 4B, C). We verified that the wild type *citS* gene rescues this defect (Figure 4C), and conclude that the nonsense mutation in *citS*, discovered by whole-genome sequencing, leads to a previously unknown defect in the citrate signal transduction pathway of the laboratory strain JH642.

Taken together, these results show that direct shotgun sequencing of the entire genome using the next generation Solexa technology is a feasible approach that also has high accuracy. This enables us to detect base substitutions that can be used to both explain and predict phenotypic differences, as demonstrated by the experimental verification with *citS*. Information on the JH642 sequence was also essential for the identification of suppressor mutations in this strain background, which is described below.

### Identification of the Molecular Nature of Spontaneous Suppressors

Based on the data obtained above, our sequencing refinement achieved the accuracy required for mapping mutations, particularly for rapidly identifying point mutations. Direct sequencing represents a simple and cost effective alternative to genetic mapping. Hence, we applied this method to identify suppressor mutations for the study of the stringent response.

**Figure 5:**
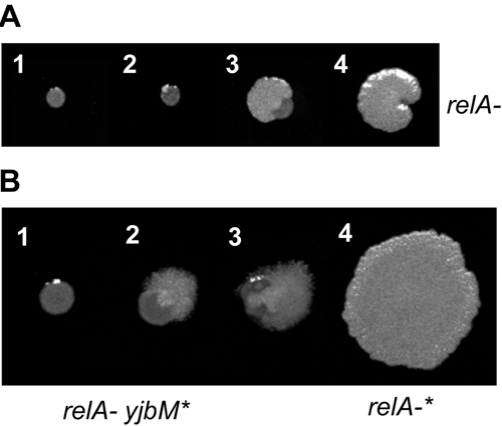
Colony morphologies of spontaneously generated suppressors. A) *relA*- colonies generate suppressors that engulf the original colonies. 1–4: gradual generation and development of suppressors. B) The yjbM* mutation partially suppresses the *relA*- growth defect, but these colonies continue to generate suppressor mutations, presumably at genomic loci other than *yjbM*. 1. *relA*- yjbM* before other suppressors are generated. 2, 3. *relA*- yjbM* with new suppressors. 4. *relA*- with double suppressors (relA- yjbM* ywaC*).

The stringent response is a ubiquitous bacterial response to starvation [9], which is mediated by the small nucleotides guanosine tetra(penta) phosphate, or (p)ppGpp. (p)ppGpp is a key factor for bacterial survival during environmental changes and is central in regulating the virulence of microbial pathogens (reviewed in [9],[29] and references therein). The *B. subtilis* bifunctional enzyme RelA modulates the intracellular levels of (p)ppGpp by both synthesizing and degrading it in response to the cellular nutritional status [30]. A *relA* deletion strain of *B. subtilis* displays a severe growth defect ([30] and Figure 5A). On plates, almost every colony eventually acquires suppressors (Figure 5A), yet none has been identified to date in *B. subtilis*. In *E. coli*, suppressors of mutations in *relA* often map to the RNA polymerase genes [9].

Using whole-genome shotgun sequencing with the Solexa technology, genomic sequences were generated for two JH642-based *relA*- strains (Figure 1E, F). One was a *B. subtilis* strain obtained by replacing the *relA* gene in JH642 with a drug resistance gene (this strain is subsequently referred to as strain *relA-*) [30]. The second strain had the same background with an identical *relA* deletion, but grew much better (on plates and in liquid media), indicating that there must be one or more second-site suppressor mutations in its genome, and hence it was named relA-*. We reasoned that since relA-* had not undergone mutagenic treatment, there should be very few sequence differences between relA-* and *relA*-. Genes in relA-* with altered bases are then likely to be good candidates for suppressors of *relA-*.

Sequencing coverage information of these two strains verified the deletion of the single *relA* gene in both strains as a 2 kb gap from 2819771 to 2821955 (Figure 1E, F and inset). In addition, in strain relA-*, a region was missing from the sequence corresponding to the 48 kb “skin element” (Figure 1F). This element interrupts a gene encoding a sporulation-specific sigma factor. When cells enter developmental phases, the “skin element” excises from the genome, allowing its flanking regions to join and form a complete gene, and thus turns on sporulation genes in the mother cell [31].

Importantly, we also identified a total of nine base changes between *relA-* and relA-*, including five missense mutations, three synonymous changes, and one intergenic change, at quality score above 40 (Table 3). There was a single point mutation in the promoter of *lexA*, the gene encoding the repressor of the SOS DNA damage response. The missense mutations were within the genes *yjbM*, *ywaC*, *yacC*, *pepT*, and *ytpQ*. Through bioinformatics analysis we noticed that the genes *yjbM* and *ywaC* shared sequence homology with *relA*. Therefore we further investigated *yjbM* and *ywaC*.

**Table 3:** Nucleotide differences between *relA*- and the *relA*-* suppressor strain.

Base Position	*relA*-	Quality	*relA*-*	Quality	Type of mutation	Gene
80,173	G	89	A	93	Missense	*yacC*
1,236,752	G	81	A	64	Missense	*yjbM*
1,522,547	T	93	C	93	Silent	*ykqC*
1,917,531	G	93	T	93	Intergenic	*lexA*
2,170,307	G	93	A	93	Silent	*yorW*
3,053,002	T	59	C	73	Missense	*ytpQ*
3,309,881	C	65	T	93	Silent	*yuxL*
3,949,118	C	93	T	93	Missense	*ywaC*
3,995,129	G	75	A	77	Missense	*pepT*

### Verification and Characterization of Suppressor Mutations and Their Interaction with *relA*

**Figure 6:**
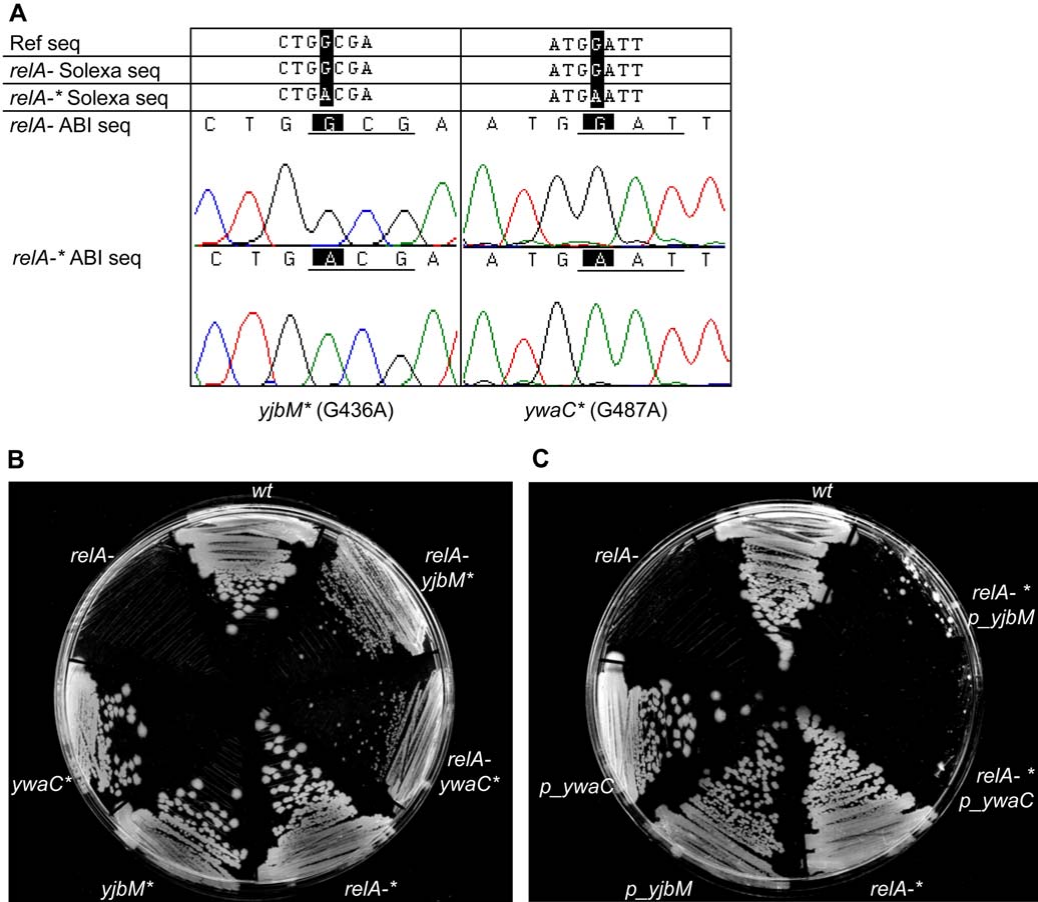
*yjbM* and *ywaC* genetically interact with *relA*. A) Verification of the Solexa-identified yjbM* and ywaC* mutations by Sanger sequencing. The reference sequence for *yjbM* or *ywaC* is shown first, followed by the Solexa results for these genes in the strain *relA-*, in which the *relA* gene is deleted, and the strain relA-* which has the identical *relA* deletion as well as second site suppressor mutations. Chromatograms of the Sanger sequencing results for each gene are shown last. B) Partial suppression of the *relA*- growth defect by introduction of either the yjbM* or ywaC* allele from *relA*-*. The strains shown in clockwise order are: i) JH642 (wt), ii) *relA-* with the yjbM* mutation from relA-* (relA-yjbM*), iii) *relA-* with the ywaC* mutation from relA-* (relA- ywaC*), iv) *relA-* with both suppressor mutations yjbM* and ywaC* (relA-*), v) yjbM* (in the JH642 background), vi) ywaC* (in the JH642 background) and vii) *relA-* (in the JH642 background, with no suppressors). The strains were plated on LB. *relA-* has a significant growth defect, which is partially relieved by introduction of either yjbM* or ywaC*, and is fully rescued by both mutations together. C) Re-introduction of a growth defect in the relA-* suppressor strain by expression of either *yjbM* or *ywaC*. The strains shown in clockwise order are: i) JH642 (wt), ii) relA-*, with suppressor mutations in both *yjbM* and *ywaC*, expressing wild type *yjbM* (*relA- p_yjbM*), iii) relA-* expressing wild type *ywaC* (relA-* p_ywaC), iv) *relA-* with suppressor mutations in *yjbM* and *ywaC* (relA-*), v) JH642 with ectopic expression of wild type *yjbM* (*p_yjbM*), vi) JH642 with ectopic expression of wild type *ywaC* (*p_ywaC*), and vii) *relA-* (in the JH642 background, with no suppressors). Full length wild type *yjbM* and *ywaC* were expressed from the IPTG-inducible *pHyperSpac* promoter and strains were plated on LB-IPTG. Expression of either *yjbM* or *ywaC* abolishes the effect of the suppressors in relA-*, and introduces a significant growth defect that is comparable to *relA-*.

In order to verify that mutations in *yjbM* (G436A) and *ywaC* (G487A) led to the suppressor phenotype, we first confirmed these sequences by the Sanger method (Figure 6A). We then separately introduced these mutations from the relA-* strain into the wild type JH642 background by standard mutation delivery methods (see Materials and Methods). The resulting two strains (annotated yjbM* and ywaC*) did not exhibit any obvious growth defect or growth advantage. But when we introduced the *relA* deletion into these two strains, both relA- yjbM* and relA- ywaC* displayed significantly improved growth on plates compared to *relA-* alone (Figure 6B), demonstrating that both were bona fide suppressor mutations.

In order to examine further the nature of the yjbM* and ywaC* mutations, we deleted either *yjbM* or *ywaC* in the *relA-* background, and found that these also suppressed the *relA*- phenotype (data not shown). We expressed the full-length wild type *yjbM* or *ywaC* genes under an inducible promoter in *relA*-* and found that there was a significant growth defect in each case (Figure 6C). Taken together, these results demonstrate that the suppressors generated in relA-* are both loss of function mutations. This was supported by an independent study which showed that loss of function mutations in *yjbM* and *ywaC* (identified by bioinformatics) alleviated the growth defect caused by deletion of *relA* [11]. In addition, YjbM and YwaC can synthesize (p)ppGpp in vitro and when introduced into *E. coli* [10],[11]. By revealing that *yjbM* and *ywaC* mutate spontaneously in response to deletion of *relA*, our results support the hypothesis that RelA acts in concert with YjbM and YwaC to modulate the cellular level of (p)ppGpp for optimal growth.

We examined how frequently *yjbM* and *ywaC* served as sites of suppressor mutations. We introduced the *relA* deletion in JH642 and sequenced independent spontaneously- generated suppressor strains, specifically at the *yjbM* and *ywaC* loci, by the Sanger method. Interestingly, 9 out of 10 new suppressors that we tested had mutations in either *yjbM* or *ywaC* (Table 4), while the one suppressor that did not have mutations in either gene had a very weakly suppressed phenotype. Among the nine strong suppressor strains, four had mutations in *ywaC* (three deletions and one base substitution) while the rest had mutations in *yjbM* (five point mutations). The mutational spectrum of suppressors included transitions, transversions, frameshifts and deletions (with or without micro-homology at the junctions).

**Table 4:** Spontaneous suppressor mutations in JH642 with a *relA* deletion.

Strain Name	*yjbM*	*ywaC*	Type of mutation	Growth&
*relA-*	wt	wt	none	−
*relA*-*	G436A	G487A	point mutations, transition	++++++
JDW483	wt	Δ(546–737)	deletion	++++
JDW484	wt	C403T	point mutation, transition	+++
JDW485	74+A	wt	insertion, frameshift	++++
JDW486	A73T	wt	point mutation, transversion	++++
JDW487	C361T	wt	point mutation, transition	++++
JDW488	C421A	wt	point mutation, transversion	++++
JDW489	wt	Δ(G610)	deletion	++++
JDW490	wt	wt	unknown	+
JDW491	A166T	wt	point mutation, transversion	++++
JDW499	wt	Δ(89–97)	deletion	++++

& estimated based on colony size.

We wondered whether the occurrence of mutations in both *yjbM* and *ywaC* in the single suppressor strain relA-* was an isolated incident, or it was because stable suppressor strains always had mutations in both *yjbM* and *ywaC*. We noticed that each suppressor mutation alone did not fully restore the growth of *relA-* cells, and the cells still exhibited a significant growth defect (Figure 6B). Consequently, the partially suppressed relA- yjbM* cells generated further suppressor mutations, as seen from colony morphologies on plates (Figure 5B). We reasoned that the secondary suppressors generated on the *yjbM* suppressor background were likely to be in *ywaC*. To test this hypothesis, we sequenced the *ywaC* region in four second-generation suppressors that spontaneously arose in relA- yjbM*, by the Sanger method. We found that all these suppressors had mutations in *ywaC* (Table 5). Two secondary suppressors had deletions in *ywaC*, while the other two had a base substitution at the *ywaC* stop codon (TAA to AAA), which potentially leads to an extended transcript that might be unstable or non-functional. Therefore, we demonstrated that most *relA*- suppressor strains, just like *relA*-*, eventually generated dual mutations in the *ywaC* and *yjbM* genes.

**Table 5:** Generation of secondary suppressor mutations in relA- yjbM*.

Strain Name	*yjbM*	*ywaC*	Type of mutation	Growth&
JDW506A	G436A	Δ(320–328)	deletion	++++++
JDW506B	G436A	T156C, Δ (88–96)	point mutation, transition deletion	++++++
JDW506C	G436A	T631A	stop codon->nonstop codon	++++++
JDW506D	G436A	T631A	stop codon->nonstop codon	++++++

& estimated based on colony size.

## Discussion

Recent advances in sequencing technologies present the opportunity to perform whole-genome sequencing of laboratory strains rapidly and at a low cost. This enables efficient detection of the genetic differences between strains at the molecular level. Furthermore, new sequencing technologies offer opportunities to develop new applications and/or to greatly simplify previously laborious experiments, such as point mutation detection. In this report, we explored a few utilities of the next generation sequencing methods for understanding the biology of a well-studied bacterium— *B. subtilis*. We demonstrated that the new shotgun-sequencing platform Solexa, which reads a large volume of short DNA fragments, can be used to obtain multiple types of genomic information from laboratory strains of *B. subtilis*. First, this technology is easily applicable for mutation detection. Genomic sequences of the widely used strains JH642, 168, SMY, and NCIB 3610 were obtained, and known mutations as well as previously unknown changes were identified. These results indicate that direct sequencing is a highly sensitive and accurate approach for detecting single base substitutions. Second, we detected large deletions of 18 kb and 9 kb, as well as deletion of a single gene (*relA*) in JH642-derived strains. Most importantly, this method enables the identification of multiple suppressor mutations in one strain and therefore provides a powerful tool to solve the often-difficult problem of suppressor identification.

### Direct Sequencing as a New Tool and its Further Enhancement

A large effort that we made while processing the sequence information was to achieve accuracy, by combining independent sequence assembly methods (MAQ, SOAP and Edena) to process reads from the Genome Analyzer. We then validated and complemented the results by sampling with Sanger sequencing and extensive experimental verification.

#### Updated Draft of the 168 Reference Sequence

Our re-sequencing of the 168 reference genome revealed ∼1800 base substitutions, in addition to insertions and deletions. We were able to test the accuracy of the Solexa reads in several independent ways and rule out Solexa sequencing errors as the likely cause of these differences. This is not surprising because the original draft was published more than a decade ago [4]. Further, we observed that the loss of isogenicity between independent isolates of the same strain was not high enough to explain the observed discrepancies. We also revealed regions with higher sequence variance, which is likely due to strain differences in the segments of DNA used in the 1997 sequencing consortium (Danchin A, personal communication). Thus we concluded that the reference sequence can be updated based on our Solexa results (DDBJ/EMBL/GenBank project accession number ABQK00000000). This version does not incorporate some heterogeneity that we observed in the ribosomal RNA operons, since these could not be mapped by just shotgun sequencing. In addition to our draft, a complete and annotated update of the previously published 168 sequence [4] is in the making and will benefit the *B. subtilis* community tremendously (Danchin A, unpublished).

#### Read Coverage and CGH

Comparative Genome Hybridization (CGH) is a microarray-based technology for studying genomic rearrangements including duplications and deletions in multiple organisms, and has been used to study DNA replication status in bacteria including *E. coli* and *B. subtilis* [32],[33]. While examining the sequence coverage, we found that direct sequencing and counting of the read coverage provide an alternative to microarray hybridization as a CGH method (Figure 1), circumventing the problems due to non-specific hybridization in microarray methods. Read coverage is the number of short DNA fragments that are read by the Genome Analyzer, and this number should be proportional to the number of DNA fragments at a specific genomic region present in the input DNA. The shape of the read coverage curve varies depending on the growth condition, and is almost flat for stationary phase cells (Figure 1A, C, D, E), indicating that these cells have completely replicated DNA, as expected. Interestingly, the coverage is slightly higher around the replication origin, and is lowest near the replication terminator, likely because a small sub-population of cells were still replicating DNA. Importantly, in an actively replicating sample (Figure 1B, F), we found a much higher read coverage near the origin (at genomic position 0/4.2 Mbp) rather than the terminus region (at genomic position 2.1 Mbp). At a higher resolution, we noticed noise in the coverage, corresponding to differing AT contents. We do not know at which step this specific enrichment of AT sequences took place; however, this can be potentially eliminated if we use the stationary phase coverage map as a reference to correct for the AT content and other unknown variations. The read coverage also gives accurate, high-resolution information on deletions, even down to the single gene level, as demonstrated by *relA*- (Figure 1E, F and inset).

#### Clustering of Mutations

Our sequencing results revealed regions with a high density of sequence variations between related strains (Figure S1). These regions could have arisen in two possible ways. First, these regions might be highly mutable. Regions of hyper-mutability have been visualized before [34] and whole-genome sequencing methods might accelerate the characterization of these changes, effectively facilitating efforts to understand the mechanisms of genomic instability, an important factor in tumorigenesis. Second and more likely, these regions might correspond to DNA of foreign origin. For example, we observed changes clustered in a 4 kb region that were likely to have arisen by horizontal gene transfer during the genetic manipulation to obtain JH642 (Figure S1). These changes are very difficult to find with traditional methods but can be easily identified by plotting the mutation distribution as shown in Figure S1. In addition, we found that the majority of the differences between 168 and SMY were located within a 6.4 kb span that includes the *trpC-D-E*, *aroH-B-F* and *cheR* genes (Figure S1). This heterogeneous cluster was identified previously by the comparison of two laboratory strains (L1437 and JH642) by microarray analysis [35],[36], and is shown to be acquired by horizontal transfer of DNA from a related *Bacillus* strain (Zeigler D, unpublished). We found that the genomic sequences of NCIB 3610 and 168 were highly similar, suggesting that they are closely related, supporting results from an independent study showing that NCIB 3610 is most likely the ancestor of 168 (Zeigler D, personal communication).

There are certain limitations to our current method. For example, while using MAQ to perform variant identification, we eliminated false positives by raising the quality score cutoff to 40. This cutoff score was chosen empirically, by shuffling and randomly dividing the Solexa sequence reads of one genome, calling the sequences independently, comparing independent calls and choosing a score that did not give any discrepancies (Figure 2). We verified that the scores obtained by MAQ were very close to the Phred scores, indicating that score 40 meant that the error rate was 0.01%. If all bases had a score of 40, we would expect ∼400 errors per genome (0.01 errors for every 100 bases of the ∼4 megabase genome). However, since most bases had scores much higher than 40, the final number of errors per genome is much closer to 0. This helped us to limit false positives so that almost all changes that we identified were bona-fide genetic differences. When this cutoff value was lowered, we obtained dramatically increased false positives. However, as a trade-off, we might not have been able to identify certain existing changes that had lower quality scores. It is also possible that the error rate we obtained might be an underestimate if there are systematic errors, although we did not identify any systematic errors while verifying our results by Sanger sequencing. In addition, although our shotgun sequencing originally identified a large number of insertions and deletions, many of these were not included in our current draft sequence since we used a high threshold to prevent the inclusion of false positives. If these changes are real, they are likely to have significant impacts including the disruption of open reading frames, which sometimes results in dominant negative or null alleles. Verification of these changes will lead to further updated versions. Similarly, certain large deletions are also not reflected in our present draft. Our current approach is also insufficient for detecting heterogeneities, such as mutation rates, in a given cell population. This is because the inherent error rate of each read is higher than the spontaneous mutation rate in cells, while each sequence call is based on the majority consensus. Despite these potential limitations, we were able to obtain a considerable number of genetic insights using whole genome shotgun sequencing. Finally, with the improvement of sequence analysis software and wider use of de novo sequence assembly programs, this method can be used to detect additional types of mutations, including DNA rearrangements.

### Genome Diversity and Phenotypic Variations between Laboratory Strains

*B. subtilis* is one of the most extensively investigated Gram-positive bacteria. Microarray-based comparative genomic hybridization (M-CGH) studies have demonstrated that there is considerable genome diversity within naturally occurring populations of *B. subtilis* strains collected from diverse geographic locations [19]. Much of the diversity was attributed to genes required for phage-related functions or those which were likely acquired by horizontal transfer. Other genes that were found to diverge significantly included those that encoded environmental sensors, detoxifying enzymes and proteins involved in antibiotic production. Essential metabolic functions were mostly encoded by less divergent genes in different populations of *B. subtilis*. Overall, as many as 28% of the genes in these strains were found to be significantly different from 168. However, between the two cultivated *B. subtilis* strains 168 and NCIB 3610, M-CGH studies revealed almost no significant sequence divergence [19]. The whole genome sequences of *B. subtilis* and its close relatives that have evolved in nature are available [37] (http://www.bacillusgenomics.org/bsubtilis).

Using whole genome sequencing to achieve near-complete coverage, we compared, base by base, the differences between related laboratory strains that have ‘evolved’ in different laboratories, and between independent isolates of several strains. We confirmed that the genomes of 168 and NCIB 3610 have few base differences, and that NCIB 3610 possesses an extra-chromosomal plasmid, that we named pAS32 [19]. We also found that individual isolates of the same strain appear to be quite isogenic, differing by only tens of bases. In particular, two different isolates of JH642 utilized in different laboratories only diverge by ∼6 bases (The actual difference between the isolates might be even smaller, since we sequenced only a single colony per isolate after streaking it out on LB plates, potentially introducing further mutations). Among these 6 variants, only 3 are missense mutations, and they are in the genes *yckJ*, *phoB and ylmF*, which encode a putative L-cystine permease, a secreted protein induced by phosphate starvation, and a hypothetical cell division protein, respectively. We have not examined the possible phenotypic differences resulting from these three missense mutations, and it remains possible that there may not be any phenotypic differences between the two isolates of JH642. Such studies provide a reasonable framework for estimating the reproducibility of experimental results obtained with independently propagated isolates.

We further discovered that several laboratory strains that are reportedly related also display tens to hundreds of base differences and insertions and deletions, including regions of horizontal transfer. Some of the variations we identified lead to phenotypic differences. For example, we discovered a novel defect in the citrate signal transduction pathway of JH642. *citS* encodes the histidine kinase sensor of a two-component system regulating the transport of citrate into *B. subtilis*. JH642, unlike its ancestral strains, has a loss of function mutation in *citS*, leading to the inability to utilize citrate as a carbon source. The revelation and our subsequent experimental verification of this defect demonstrate the power of whole-genome sequencing.

### Tripartite Genetic Interaction between (p)ppGpp Synthases in *B. subtilis*

*B. subtilis* is a powerful model system to identify genetic pathways. One common approach to identify components of a given genetic pathway is through genetic modifier screens- enhancer and suppressor screens. However, identification of the molecular nature of the mutations obtained in a genetic screen is often laborious. Furthermore, in some cases, it can be difficult to identify mutations using traditional genetic mapping; for example, in the absence of an expression library or when the phenotype observed is due to the combinatorial effect of multiple mutations rather than one mutation alone. The potential difficulty due to multiple suppressors can be easily resolved with whole-genome sequencing, as we have demonstrated by identifying in a single strain, two *relA*- suppressor mutations in the *relA* homologs *yjbM* and *ywaC*.

**Figure 7:**
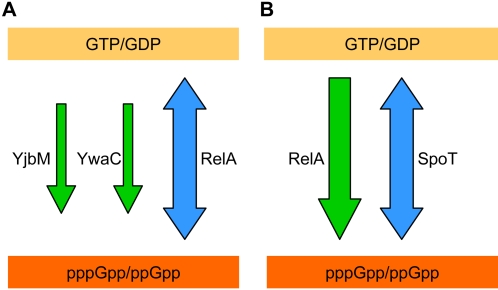
Metabolism of (p)ppGpp. A). Metabolism of (p)ppGpp in *B. subtilis*. (p)ppGpp is synthesized by the enzymes YjbM, YwaC and RelA, but degraded only by RelA. B). Metabolism of (p)ppGpp in *E. coli*. (p)ppGpp is synthesized by the enzymes RelA and SpoT, but degraded only by SpoT.

In *B. subtilis*, the pre-existing paradigm for stringent control was that a single synthase/hydrolase of (p)ppGpp, the RelA protein, modulated the stringent response to nutritional stress [30]. Using whole-genome sequencing, we found that within one *B. subtilis relA-* strain, two suppressor mutations spontaneously arose, each mapping to a different homolog of *relA* and contributing to the partial recovery of growth. Multiple suppressors of *relA-* which are generated independently and spontaneously had mutations that mapped almost exclusively to *ywaC* and *yjbM*. These two small homologs of RelA were independently identified using bioinformatics approaches in *Streptococcus mutans* and *B. subtilis* and possess only the synthesis, but not the hydrolysis and regulatory activities of RelA [10],[11]. Our results demonstrate strong genetic interactions among the three genes, and that RelA, rather than acting alone, acts in concert with these two other (p)ppGpp synthases (Figure 7A). Deletion of *relA* abolishes the cells' ability to degrade (p)ppGpp, thus leading to poor growth likely because they produce too much (p)ppGpp rather than too little. This growth defect might subsequently trigger mutations in *yjbM* and *ywaC*, which encode (p)ppGpp synthases. Finally, the strain evolves to eliminate (p)ppGpp synthesis activity, and is not as viable as a wild type strain that has all three genes, but nonetheless attains a strong growth advantage with respect to the *relA-* strain. Intriguingly, (p)ppGpp is virtually undetectable both in the relA-* suppressor strain and the *relA-* deletion strain (data not shown) [30], by thin layer chromatography (TLC), likely because *relA-* cells possess levels of (p)ppGpp that are below the limit of detection of TLC. In addition, within a population of *relA-* cells, individual cells that accidentally produce (p)ppGpp will not be able to degrade it and therefore will fail to grow and divide, resulting in a further diluted level of the nucleotide in a population. Our results do not rule out the possibility that RelA interacts directly with YjbM and/or YwaC to modulate their function and prevent any deleterious effects caused by their unregulated activity.

A comparison can be made with *E. coli*, where (p)ppGpp is synthesized by two proteins, RelA and SpoT (Figure 7B). RelA produces (p)ppGpp, and SpoT can both produce and hydrolyze (p)ppGpp. The *spoT*- strain is not viable and can only be relieved by *relA*- mutations, while *relA*- alone is viable. Similarly, in *B. subtilis*, *relA-* is relieved by *yjbM-* and *ywaC-* mutations. *B. subtilis* differs from *E. coli* in having two enzymes that purely synthesize (p)ppGpp (Figure 7A). Therefore, loss of function of either enzyme alone is not sufficient to relieve the effect of loss of RelA hydrolase activity. Our experiments support an emerging paradigm that Gram-positive bacteria utilize three enzymes for (p)ppGpp production and/or degradation, all of which perhaps play important roles in bacterial stress responses [10],[11].

The tripartite genetic network that controls (p)ppGpp levels determines the evolutionary landscape that leads to the generation of multiple suppressors. Conversely, the pathways that generate suppressors can reveal the evolutionary landscape of an organism and subsequently illuminate its cellular infrastructure [38],[39]. The vast majority of *relA*- suppressor strains have mutations in *yjbM* or *ywaC*, and almost all colonies eventually develop mutations in both genes. The occurrence of dual mutations is likely due to strong evolutionary pressure for increased fitness, and hence is a natural consequence of the tripartite regulation. The nature of this evolutionary landscape supports genetic interactions that involve three loci, instead of the more traditional module of two loci like *spoT* and *relA* in *E. coli*. Similar regulatory networks involving more than two gene loci are likely to be more common than previously believed and whole-genome sequencing is a powerful tool to uncover such systems.

Close examination of the molecular nature of the suppressive genomic changes indicates that there is no obligatory cascade of mutagenic events that is triggered by *relA* deletion (Tables 4, 5). Diverse types of mutations arise, including insertions, deletions, and different types of point mutations (both transitions and transversions), which are likely mediated by different mechanisms. We did notice that mutations in *ywaC* seemed to involve a higher incidence of deletions, although larger sequencing-based sampling is required before a conclusion can be drawn. Alleviation of the *relA-* growth defect does not require concurrent mutations in *yjbM* and *ywaC*, but can be achieved by sequential inactivation of these genes (Figure 5B). This result confirms the ability of bacteria to manipulate their genomes quickly to generate mutations that counter an unfavorable genetic change. The process of stress-induced mutagenesis is likely behind this plasticity [40]. Intriguingly, (p)ppGpp is strongly implicated in the mechanism of stress-induced mutagenesis and it is thought that genes whose transcription is up-regulated by (p)ppGpp are more susceptible to (p)ppGpp-induced mutagenesis [41],[42]. Are *yjbM* and *ywaC* such genes and therefore specifically targeted for mutagenesis? What are the respective roles of YjbM, YwaC and RelA in sensing separate environmental stresses? These are intriguing questions that remain to be elucidated.

## Materials and Methods

### Strains, Media, and Growth Conditions

*B. subtilis* strains used are listed in Table 2. JH642 was obtained from the Bacillus Genetic Stock Center (BGSC) (1A96) and from A.D. Grossman (BGSC 1A867). *B. subtilis* 168 (BGSC 1A1 and 1A700) and NCIB 3610 (BGSC 3A1) were obtained from the BGSC. SMY was obtained from the BGSC (1A775) and A.L. Sonenshein (BGSC 1A868). Genetic manipulations were performed using standard protocols [43]. Erythromycin and lincomycin (MLS) were used at 5 µg/ml [43] for *relA*- strains. Strains were grown with vigorous shaking at 37°C in LB medium. To isolate suppressors, freshly transformed *relA*- colonies were inoculated in individual tubes of LB and cells were grown to saturation and plated by serial dilutions. One large colony on each plate was selected and its DNA sequenced by the Sanger method.

### Gene Replacement in JH642

PCR was conducted using genomic DNA from NCIB 3610 to amplify the *pheA and trpC* genes. The *pheA* PCR product was gel-purified and used to transform JH642. Transformants were selected on minimal plates supplemented with glucose and tryptophan to obtain JDW441. The *trpC* PCR product was gel purified and used to transform JDW441. Transformants were selected on minimal plates supplemented with glucose as the sole carbon source to obtain JDW442. The *citS* gene was amplified from SMY, then gel-purified and used to transform JDW442. Transformants were selected on minimal plates supplemented with citrate as the sole carbon source to obtain JDW522.

### Mutation Delivery

PCR was conducted using relA-* genomic DNA and the primers oJW212/213 to amplify yjbM*. The PCR products were digested with EcoRI and cloned into pEX44. The resulting plasmid, pAT001, was used to transform JH642 and chloramphenicol (Cm) resistant transformants were selected, resulting in strain AT004. AT004 was grown overnight without selection and plated on X-gal (40 µg/ml). Cm sensitive colonies were isolated and sequenced to verify that they contained the yjbM* mutation, to obtain JDW506. For ywaC*, similar procedures were employed using primers oJW215/218 and EcoRI/BamHI for digestion, resulting in AS021.

oJW212: CCCGAATTCGAAACGCGTGTTTTTATAGAAACTG

oJW213: CCCGAATTCTAAAAA CCCAGATGACCTGT

oJW215: CCCGGATCCAGGATCATCTGATCATGTGC

oJW218: GCGGATAAAGTTCCGTTAAAGGAGA

### Genetic Interaction Analysis

The *yjbM* and *ywaC* genes were amplified from JH642 genomic DNA using PCR primers oJW229/230 and oJW231/232 respectively, and introduced into the SphI-SalI sites of the vector pDR90. The resulting plasmids pAS001 and pAS002 were used to transform JH642 to spectinomycin resistance by homologous recombination at the *amyE* locus, giving rise to strains AS012 and AS013. Genomic DNA from AS012 and AS013 were used to transform the strain relA-*, and spectinomycin-resistant transformants were selected, to obtain relA-* p_yjbM and relA-* p_ywaC, respectively. Genomic DNA from *relA-* was used to transform JDW506 and AS021, and MLS-resistant transformants were selected to obtain relA- yjbM* and relA- ywaC*, respectively. To compare growth, these strains were plated to obtain single colonies on LB or LB-IPTG plates (1 mM IPTG).

oJW229: GCTGGTCGACATTGGGGGATGTATGATGG

oJW230: GTTGGCATGCTCATGCTCTTCTTCCCCTTT

oJW231: GCTGGTCGACTCCGTTAAAGGAGATGACGAA

oJW232: GTTGGCATGCACTTGGGTGCCGTCTTTTT

### Solexa Sequencing and Analysis of Sequences

Genomic DNA was purified using the Qiagen Genomic DNA Purification kit. Shotgun DNA libraries were generated according to the manufacturer's sample preparation protocol for genomic DNA. Briefly, 1–5 µg genomic DNA was randomly sheared using nebulizers and the ends were repaired using polynucleotide kinase and Klenow enzymes. The 5′ ends of the DNA fragments were phosphorylated and a single adenine base was added to their 3′ ends using Klenow exo^+^. Following ligation of a pair of Solexa adaptors to the repaired ends, the DNA was amplified using adaptor primers for 18 cycles, and fragments around 150 bp long were isolated from agarose gels. Sequencing libraries were quantified with an Agilent 2100 Bioanalyzer as well as a picogreen fluorescence assay. Cluster generations were performed on an Illumina cluster station using 2 pM sequencing libraries. 36 cycles of sequencing were carried out using the Illumina/Solexa Genome Analyzer system according to the manufacturer's specifications.

Sequencing analysis was first done using the Solexa analysis pipeline. On average, about 2.5 million successful reads, consisting of 36 bases of each fragment, were generated on each lane of a flow cell. The output of the Solexa Analysis Pipeline was fed into the third-party software MAQ (Mapping and Assembly with Qualities, http://maq.sourceforge.net, Heng Li, Richard Durbin). Reads were aligned (mapped) to the published complete genome for *Bacillus subtilis subsp. subtilis str.* 168 (GenBank: AL009126). At the mapping stage, MAQ performs un-gapped alignment of the Solexa sequence reads to the reference sequence. At the assembly stage, MAQ uses a statistical model to generate the consensus. It calls the bases that maximize the Bayesian posterior probability, and calculates a phred quality at each position along the consensus, which is logarithmically linked to the error probability. The consensus quality scores were used in this manuscript. In addition, the algorithm SOAP (Short Oligonucleotide Alignment Program) [14] was used to iteratively map all the reads to the reference genome with parameters that allowed gaps and up to 3 mismatches (-v 3 –g 5 –c43). Finally, de novo assembly was conducted with the Edena software (Exact De Novo Assembler) [15], using its default parameters. The assembled contigs were aligned to the reference genome using the *Crossmatch* program that is based on the Smith-Waterman alignment algorithm [44]. Ad hoc programs developed in the laboratory were used to perform shuffle experiments, mutation detection and annotation, and genomic sequence comparisons between strains.

## Supporting Information

Figure S1 The distribution of mutations reveals clusters of heterologous genes.
(1.57 MB TIF)

Table S1 Bases that are different between the two isolates of *B. subtilis* strain 168 (BGSC 1A1 and 1A700).
(0.02 MB XLS)

Table S2 Bases that are different between the two isolates of *B. subtilis* strain SMY (BGSC 1A868 and 1A775).
(0.02 MB XLS)

Table S3 Bases that are different between the two isolates of *B. subtilis* strain JH642 (BGSC 1A867 and 1A96).
(0.02 MB XLS)

## References

[1] Sonenshein AL, Hoch JA, Losick R, Sonenshein AL, Hoch JA, Losick R (2002). *Bacillus subtilis* and its closest relatives: from genes to cells;.

[2] Burkholder PR, Giles JHN (1947). Introduced biochemical mutations in *Bacillus subtilis*.. Am J Bot.

[3] Spizizen J (1958). Transformation of Biochemically Deficient Strains of *Bacillus subtilis* by Deoxyribonucleate.. Proc Natl Acad Sci U S A.

[4] Kunst F, Ogasawara N, Moszer I, Albertini AM, Alloni G (1997). The complete genome sequence of the gram-positive bacterium *Bacillus subtilis*.. Nature.

[5] Albertini AM, Galizzi A (1999). The sequence of the *trp* operon of *Bacillus subtilis* 168 (trpC2) revisited.. Microbiology.

[6] Brehm SP, Staal SP, Hoch JA (1973). Phenotypes of pleiotropic-negative sporulation mutants of *Bacillus subtilis*.. J Bacteriol.

[7] Dean DR, Hoch JA, Aronson AI (1977). Alteration of the *Bacillus subtilis* glutamine synthetase results in overproduction of the enzyme.. J Bacteriol.

[8] Wiegeshoff F, Marahiel MA (2007). Characterization of a mutation in the acetolactate synthase of *Bacillus subtilis* that causes a cold-sensitive phenotype.. FEMS Microbiol Lett.

[9] Cashel M, Gentry DR, Hernandez VH, Vinella D, Neidhardt FC, Curtiss R, Ingraham JL, Lin ECC, Low KB, Magasanik B, Reznikoff WS, Riley M, Schaechter M, Umbarger HE (1996). The stringent response.. *Escherichia coli* and *Salmonella*: cellular and molecular biology. 2nd ed.

[10] Lemos JA, Lin VK, Nascimento MM, Abranches J, Burne RA (2007). Three gene products govern (p)ppGpp production by *Streptococcus mutans*.. Mol Microbiol.

[11] Nanamiya H, Kasai K, Nozawa A, Yun CS, Narisawa T (2008). Identification and functional analysis of novel (p)ppGpp synthetase genes in *Bacillus subtilis*.. Mol Microbiol.

[12] Hillier LW, Marth GT, Quinlan AR, Dooling D, Fewell G (2008). Whole-genome sequencing and variant discovery in *C. elegans*.. Nat Methods.

[13] Ewing B, Green P (1998). Base-calling of automated sequencer traces using phred. II. Error probabilities.. Genome Res.

[14] Li R, Li Y, Kristiansen K, Wang J (2008). SOAP: short oligonucleotide alignment program.. Bioinformatics.

[15] Hernandez D, Francois P, Farinelli L, Osteras M, Schrenzel J (2008). De novo bacterial genome sequencing: Millions of very short reads assembled on a desktop computer.. Genome Res.

[16] Schaeffer P, Millet J, Aubert JP (1965). Catabolic repression of bacterial sporulation.. Proc Natl Acad Sci U S A.

[17] Hoch JA, Mathews JL (1973). Chromosomal location of pleiotropic negative sporulation mutations in Bacillus subtilis.. Genetics.

[18] Perego M, Spiegelman GB, Hoch JA (1988). Structure of the gene for the transition state regulator, *abrB*: regulator synthesis is controlled by the *spo0A* sporulation gene in *Bacillus subtilis*.. Mol Microbiol.

[19] Earl AM, Losick R, Kolter R (2007). *Bacillus subtilis* genome diversity.. J Bacteriol.

[20] Tanaka T, Ogura M (1998). A novel *Bacillus natto* plasmid pLS32 capable of replication in *Bacillus subtilis*.. FEBS Lett.

[21] Itaya M (1993). Stability and asymmetric replication of the *Bacillus subtilis 168* chromosome structure.. J Bacteriol.

[22] Lopez-Torrejon G, Martinez-Jimenez MI, Ayora S (2006). Role of LrpC from *Bacillus subtilis* in DNA transactions during DNA repair and recombination.. Nucleic Acids Res.

[23] DiGate RJ, Marians KJ (1989). Molecular cloning and DNA sequence analysis of *Escherichia coli topB*, the gene encoding topoisomerase III.. J Biol Chem.

[24] Kobayashi K, Ehrlich SD, Albertini A, Amati G, Andersen KK (2003). Essential *Bacillus subtilis* genes.. Proc Natl Acad Sci U S A.

[25] Castellanos-Juarez FX, Alvarez-Alvarez C, Yasbin RE, Setlow B, Setlow P (2006). YtkD and MutT protect vegetative cells but not spores of *Bacillus subtilis* from oxidative stress.. J Bacteriol.

[26] Maki H, Sekiguchi M (1992). MutT protein specifically hydrolyses a potent mutagenic substrate for DNA synthesis.. Nature.

[27] Tsuge K, Ano T, Hirai M, Nakamura Y, Shoda M (1999). The genes *degQ*, *pps*, and *lpa-8* (*sfp*) are responsible for conversion of *Bacillus subtilis 168* to plipastatin production.. Antimicrob Agents Chemother.

[28] Yamamoto H, Murata M, Sekiguchi J (2000). The CitST two-component system regulates the expression of the Mg-citrate transporter in *Bacillus subtilis*.. Mol Microbiol.

[29] Srivatsan A, Wang JD (2008). Control of bacterial transcription, translation and replication by (p)ppGpp.. Curr Opin Microbiol.

[30] Wendrich TM, Marahiel MA (1997). Cloning and characterization of a *relA*/*spoT* homologue from *Bacillus subtilis*.. Mol Microbiol.

[31] Kunkel B, Losick R, Stragier P (1990). The *Bacillus subtilis* gene for the development transcription factor sigma K is generated by excision of a dispensable DNA element containing a sporulation recombinase gene.. Genes Dev.

[32] Wang JD, Sanders GM, Grossman AD (2007). Nutritional control of elongation of DNA replication by (p)ppGpp.. Cell.

[33] Khodursky AB, Peter BJ, Schmid MB, DeRisi J, Botstein D (2000). Analysis of topoisomerase function in bacterial replication fork movement: use of DNA microarrays.. Proc Natl Acad Sci U S A.

[34] Drake JW, Bebenek A, Kissling GE, Peddada S (2005). Clusters of mutations from transient hypermutability.. Proc Natl Acad Sci U S A.

[35] Berkmen MB, Grossman AD (2007). Subcellular positioning of the origin region of the *Bacillus subtilis* chromosome is independent of sequences within oriC, the site of replication initiation, and the replication initiator DnaA.. Mol Microbiol.

[36] Wang JD, Berkmen MB, Grossman AD (2007). Genome-wide coorientation of replication and transcription reduces adverse effects on replication in *Bacillus subtilis*.. Proc Natl Acad Sci U S A.

[37] Earl AM, Losick R, Kolter R (2008). Ecology and genomics of *Bacillus subtilis*.. Trends Microbiol.

[38] Lenski RE, Travisano M (1994). Dynamics of adaptation and diversification: a 10,000-generation experiment with bacterial populations.. Proc Natl Acad Sci U S A.

[39] Poelwijk FJ, Kiviet DJ, Weinreich DM, Tans SJ (2007). Empirical fitness landscapes reveal accessible evolutionary paths.. Nature.

[40] Galhardo RS, Hastings PJ, Rosenberg SM (2007). Mutation as a stress response and the regulation of evolvability.. Crit Rev Biochem Mol Biol.

[41] Wright BE (1996). The effect of the stringent response on mutation rates in *Escherichia coli* K-12.. Mol Microbiol.

[42] Rudner R, Murray A, Huda N (1999). Is there a link between mutation rates and the stringent response in *Bacillus subtilis*?. Ann N Y Acad Sci.

[43] Harwood CR, Cutting SM (1990). Molecular biological methods for *Bacillus*.

[44] Smith TF, Waterman MS (1981). Identification of common molecular subsequences.. J Mol Biol.

