# Supplementary figures and images for: High-Precision, Whole-Genome Sequencing of Laboratory Strains Facilitates Genetic Studies
Source: PLoS Genet. 2008 Aug 1;4(8):e1000139. doi: 10.1371/journal.pgen.1000139 (PMC2474695; doi:10.1371/journal.pgen.1000139)

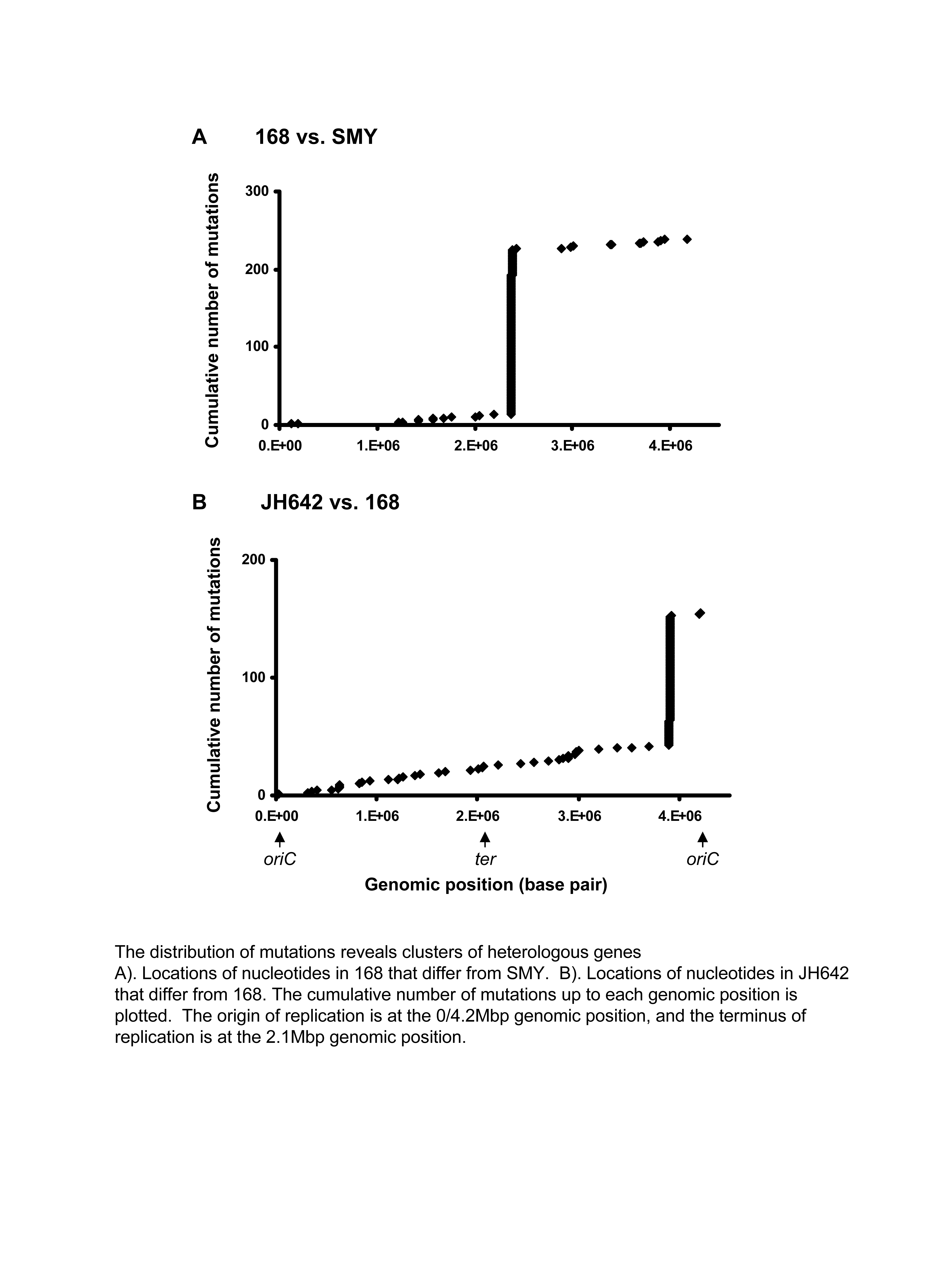

Supplement: Figure S1 — The distribution of mutations reveals clusters of heterologous genes. (1.57 MB TIF) [file pgen.1000139.s001.tif]
